# Latitudinal diversity in circadian and light-sensing genes in an ecologically vital group of marine picoeukaryote algae

**DOI:** 10.1093/ismejo/wraf263

**Published:** 2025-11-28

**Authors:** Janaina Rigonato, Jean-Claude Lozano, Valérie Vergé, Olivier Jaillon, Francois-Yves Bouget

**Affiliations:** Laboratoire d'Océanographie Microbienne (LOMIC), CNRS/Sorbonne Université, UMR 7621, Observatoire Océanologique, 1 Av. Pierre Fabre, 66650 Banyuls-sur-Mer, France; Génomique Métabolique, Genoscope, Institut François Jacob, CEA, CNRS, Univ Evry, Université Paris-Saclay, 2 Rue Gaston Crémieux, 91000 Évry-Courcouronnes, France; Laboratoire d'Océanographie Microbienne (LOMIC), CNRS/Sorbonne Université, UMR 7621, Observatoire Océanologique, 1 Av. Pierre Fabre, 66650 Banyuls-sur-Mer, France; Laboratoire d'Océanographie Microbienne (LOMIC), CNRS/Sorbonne Université, UMR 7621, Observatoire Océanologique, 1 Av. Pierre Fabre, 66650 Banyuls-sur-Mer, France; Génomique Métabolique, Genoscope, Institut François Jacob, CEA, CNRS, Univ Evry, Université Paris-Saclay, 2 Rue Gaston Crémieux, 91000 Évry-Courcouronnes, France; Laboratoire d'Océanographie Microbienne (LOMIC), CNRS/Sorbonne Université, UMR 7621, Observatoire Océanologique, 1 Av. Pierre Fabre, 66650 Banyuls-sur-Mer, France

**Keywords:** phytoplankton, circadian clock, photoreceptors, photoperiodism, mamiellophyceae

## Abstract

Organismal life cycles are influenced by Earth’s rotation and orbit, generating daily and seasonal light cycles that vary with latitude, especially in temperate and polar zones. Photoperiodism relies on organisms’ ability to measure time via the circadian clock and detect light through specific photoreceptors. Molecular basis of photoperiodism is well-characterized in plants, but photoperiod adaptation in phytoplankton remain largely unexplored. Here, we investigated circadian clock components, photoreceptors, and associated effectors in eukaryote picoalga species from *Ostreococcus*, *Bathycoccus*, and *Micromonas*. We showed that the investigated species shared a conserved set of homologous circadian clock-related genes that appeared in the early evolution of Mamielalles order. Furthermore, gene duplication events account for the specific occurrences and uneven gene copy numbers among these genera. Through metagenomic and metatranscriptomic analyses, we assessed the gene expression profiles of candidate photoperiod-related genes across the global ocean. Our findings reveal an unexpected diversity in photoreceptors, particularly within *Micromonas*, and highlight the CCT domain family, a key group of transcription factors governing circadian rhythms (TOC1 family) and photoperiodism (CONSTANS family) in plants. TOC1, a central component of the circadian clock in *Ostreococcus tauri*, is either absent or truncated in tropical species. Functional assays further indicate that the TOC1/CCA1 oscillator is nonfunctional in the tropical strain of *Ostreococcus* sp. RCC809. These results imply that certain circadian mechanisms may be dispensable at low latitudes, underscoring the diversity of photoperiod adaptations in marine phytoplankton. These results provide valuable insights into the molecular evolution of cosmopolitan plankton groups, particularly their mechanisms of local adaptation.

## Introduction

Marine phytoplankton contributes significantly to global primary production, at levels comparable to terrestrial plants, and inhabit a broad range of latitudinal niches from polar to equatorial regions [1]. Recent advances in large-scale metagenomics surveys from oceanic expeditions have revealed the great diversity of both prokaryotic and eukaryotic phytoplankton, with temperature emerging as a primary factor shaping niche differentiation [2, 3]. In temperate oceanic regions, phytoplankton often form seasonal blooms influenced by temperature and photoperiod [4].

Optimizing light utilization in response to seasonal changes in photoperiod is crucial for terrestrial plants and microalgae, which rely on light to convert carbon dioxide into organic carbon for growth and reproduction. In plants, seasonal life cycle events, most in particular the timing of flowering in short-day and long-day species, are primarily regulated by day length, a phenomenon known as photoperiodism [5]. Nevertheless, some microalgal groups, such as dinoflagellates and Mamiellophyceae, display marked seasonal patterns; e.g. dinoflagellates activate resting cells in response to seasonal cues [4, 6]. Moreover, long-term time-series studies at coastal sites, where environmental conditions such as temperature fluctuate markedly, indicate that photoperiod is a major driver of phytoplankton phenology [7]. These findings suggest that intrinsic timekeeping mechanisms, similar to those in plants, may also regulate seasonal dynamics in marine phytoplankton.

Living organisms have developed sophisticated timekeeping mechanisms to orchestrate biological processes with daily and seasonal environmental cycles [8, 9]. At the core of this synchronization lies the circadian clock, found across nearly all life forms [8]. The circadian clock orchestrates not only global transcriptional networks along day/night cycles but also post-transcriptional responses that sustains essential metabolic processes [10]. Although less well documented than in the freshwater cyanobacterium *Synechococcus elongatus*, marine *Synechococcus* spp. exhibit circadian rhythms primarily governed by the protein-based KaiABC phosphorylation cycle [11]. Eukaryotic marine phytoplankton circadian clock is presumed to operate via transcription–translation feedback loops. In the diatom *Phaeodactylum tricornutum* the bHLH–PAS transcription factor RITMO1 acts as a component of the circadian clock [12, 13].

**Figure 1:**
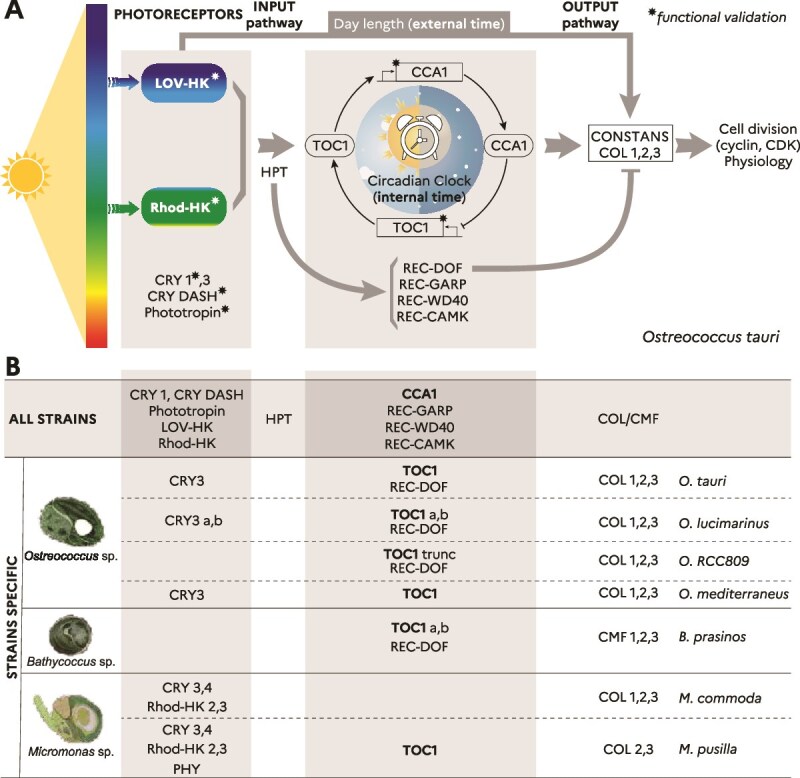
Comparative analysis of circadian clock and light sensing candidates in Mamiellophyceae. (A) Schematic representation of the circadian clock system in *O. tauri,* illustrating the key light input components, oscillator and putative output pathways. Oscillator core genes include the transcriptional activator timing of CAB expression1 homologue (*TOC1*) and its transcriptional repressor Circadian Clock Associated 1 homologue (*CCA1*). Photoreceptors include blue light and green light LOV-histidine (LOV-HK) and Rhodopsin histidine kinases (RHOD-HK) possibly mediating qualitative light cues to TOC1 through a two component system histidyl-aspartyl signalling pathway, which may involve an histidine Phosphotransfer (HPT) protein. Four other putative response regulators, similar to TOC1, contain a functional receiver (REC) domain associated with various DNA-binding or regulatory domains. These include the DNA-binding with one finger (DOF) domain, a known regulator of CONSTANS; the GARP transcription factor domain, (found in CONSTANS, Golden2, ARR-B, and Psr1,) commonly associated with plant circadian clock and photoperiodism function; as well as the calcium/calmodulin-dependent protein kinase (CaMK) and the β-propeller WD40 domains. CONSTANS-like (COL/CMF) candidates are hypothesized to be key elements in the output pathway that integrates circadian oscillator signals and photoreceptor-mediated light signals to modulate photoperiodic responses. ^*^symbol indicate genes that have been experimentally validated with a circadian clock and/or photoreceptor function. (B) List of genes identified in this study for the different species of Mamiellophyceae (for details see Tables S2 and S3). TOP “all strains”: Genes present in all analysed genomes. BOTTOM “strains specific”: Genes specific to one or several species.

Among green picoalgae, *Ostreococcus tauri* (Mamiellophyceae) has emerged as a key model for understanding eukaryotic circadian regulation [14]. *Ostreococcus tauri* possesses a Green lineage type of circadian system comparatively simpler than that of plants [14]. In this species, cell division, iron metabolism, and more generally global transcription patterns are strongly regulated by day/night cycles [15–17]. The *O. tauri* circadian clock relies on the master genes Circadian Clock Associated 1 (*CCA1*) and Timing of CAB Expression 1 (*TOC1*), which were identified by the presence of protein functional domains conserved with plants, namely MYB/SANT for CCA1; receiver (REC) and CCT (for CONSTANS, CONSTANS-like and TOC1) for TOC1 (Fig. 1A) [14]. The TOC1, a pseudo-response regulator (PRR), activates *CCA1*, whereas CCA1 represses *TOC1* expression by binding to the conserved Evening Element in its promoter [14, 16]. In addition, *O. tauri* encodes several photoreceptors that provide light input to the circadian system. These include Light-Oxygen-Voltage histidine kinase (LOV-HK) and various Cryptochromes, such as animal like CRY1 and CRY DASH, identified as blue light photoreceptors [18, 19]*.* A second type of sensory histidine kinase with a Rhodopsin domain (Rhod-HK) function as a green light photoreceptor, and together with LOV-HK, may measure and transduce the blue-to-green light ratio, thereby acting as both photoperiod and depth sensors through downstream response regulators (RRs) [20, 21]. Phototropin, a plant blue light photoreceptor containing a LOV domain fused to a serine threonine kinase, is also present in *O. tauri* (Fig. 1A) [22].

Although the downstream pathways integrating circadian and photoperiodic signals remain unknown in *O. tauri*, two putative regulators of plant photoperiodism CONSTANS-like (COL1 and COL2) have been identified based on the presence of zinc-finger B-box and CCT domains [14]. In *Arabidopsis*, CONSTANS is transcriptionally regulated by the circadian clock, but the CONSTANS protein is degraded during the night, promoting flowering under long-day conditions, in agreement with the external coincidence model [23]. Variations in the CCT domain proteins (for CONSTANS, CONSTANS-like and TOC1) have been associated with adaptation to specific latitudes in domesticated cereals such as rice, barley, sorghum, millet, maize, and soybean [24–27], providing a valuable resource for identifying genetic determinants of photoperiodic responses under short- or long-day conditions. Additionally, the CONSTANS-like protein from the green alga *Chlamydomonas reinhardtii* can complement the *Arabidopsis co* mutant when expressed heterologously, indicating functional conservation across lineages [28]. This observation raises the possibility that CONSTANS may also be involved in photoperiodic regulation in *O. tauri*.

Although the general architecture of the circadian clock and its associated photoreceptors is now reasonably well understood in *O. tauri*, how these light sensing and timekeeping systems are involved in adaptation to varying photoperiod in the ocean remains unclear. Recent study on the tropical strain *Ostreococcus* sp. RCC809 has shown that this low-latitude species exhibits a unique response to light quality, distinguishing it from the shallow lagoon species *O. tauri* [29]. In our study, we explored the functional diversity of light-sensing and circadian clock components by analyzing genomes and metagenomic data from related Mamiellales species (*Bathycoccus prasinos*, *Ostreococcus* spp., and *Micromonas* spp*.*) across the global ocean.

## Material and methods

### Genomic analysis of marine Picoalgae in the order Mamiellales

In this study, we analyzed annotated genomic data from marine picoalgae belonging to the order Mamiellales, available through the Online Resource for Community Annotation of Eukaryotes (ORCAE*—*Table S1) [30]. Homologs of circadian clock and photoreceptor sequences previously identified in *O. tauri w*ere also found in the predicted protein datasets of *Bathycoccus prasinos, Ostreococcus lucimarinus*, *Ostreococcus mediterraneus*, *Ostreococcus* sp*.* RCC809, *Micromonas commoda*, and *Micromonas pusilla* using BLASTP [31].

Conservation of amino acid sequences was generally sufficient to identify putative homologs within th*e Ostreococcus* spp*.* using BLASTP (e.g. in the case of TOC1, E-value <2 × 10^−100^ and amino acid identity >50% over the full protein length*).* However, for *Bathycoccus prasinos* and *Micromonas* spp., sequence conservation was mostly limited to conserved protein functional domains. To address this, we extended our analysis to coding sequences (CDSs) of all surveyed genomes, focusing on domains associated with light/photoperiod sensing and circadian clock regulation, using the ExPASy PROSITE tool [32]*.* A protein was considered a putative homolog of an *O. tauri* candidate when all expected functional domains were predicted (e.g. REC and CCT domains for TOC1). The identified proteins, their associated domains and corresponding genes are listed in Tables S2 and S3. In a few cases, we recovered only partial protein sequences due to inaccuracies in CDS annotations, often caused by incorrect predictions of intron–exon boundaries. This issue was particularly evident in the CONSTANS-like family, where an intron spanning the B-box domain was mispredicted in most CONSTANS-like sequences. To address this, *O. tauri* protein sequences were queried against the genomic nucleotide sequences of the evaluated species using the tBLASTn algorithm [33] and the annotations were manually corrected, enabling the recovery of full-length gene models and re-annotation of the corresponding CDS (Tables S2 and S3). In addition to TOC1, our search for receiver domains revealed four additional candidates in *O. tauri* likely involved in two-component signalling pathways. These candidates contained domains such as DOF, GARP, WD40, or Calmodulin Kinase (CAMK) associated with the REC domain (Tables S2 and S3).

### Phylogeny

Protein sequences of circadian clock components recovered from the investigated strains (Table S3) were aligned using Clustal Omega [34] implemented in Geneious Prime v. 2023.2.1 software. To enhance alignment accuracy, we annotated protein domains for each sequence prior to alignment, and we employed an iterative alignment strategy (refined iterations = 5) to improve alignment accuracy in divergent regions while preserving conserved domains. Resulting alignments were manually inspected to ensure optimal quality. Phylogenetic trees were constructed using Maximum Likelihood criterion performed with RAxML method [35] and GAMMA DAYHOFF protein model, also implemented in Geneious Prime. The most likely tree, selected from 500 bootstrap replicates, was chosen to represent the inferred phylogeny. To minimize artifacts caused by highly divergent outgroups, midpoint rooting was applied, placing the root at the midpoint of the longest path between two sequences. This approach provides a practical and unbiased solution for visualizing phylogenetic relationships while reducing distortions from uneven evolutionary rates [36].

A 18S rRNA phylogenetic tree was built to facilitate the comprehension of the circadian clock genes phylogeny discussion. The 18S rRNA sequences in the different genomes were identified by BLASTn on ORCAE portal. Additionally, sequences from other genera of Mamiellophyceae Class was also retrieved from GenBank data base, to construct the tree. *C. reinhardtii* was elected as outgroup.

### Environmental metagenome and metatranscriptome screening

To investigate the distribution and variation of circadian clock genes and photoreceptors in the oceans, we recruited metagenomics reads obtained by the Tara Oceans (TOs) and Tara Oceans Polar Circle (TOPC) projects [37, 38]. Metagenomic data (MetaG) were mapped onto the reference genome of each investigated strain (Table S1) individually using BWA mem 0.7.17 with default parameters [39]. The resulting alignments were converted to “.bam” format and underwent filtering to eliminate unmapped reads, and sorting using Samtools 1.13 [40] applying the following steps: (i) filtering to remove unmapped reads (view -F 4), (ii) sorting (sort), and (iii) duplicate removal (rmdup) (https://github.com/samtools/samtools). Metatranscriptomic data (MetaT) were aligned to the reference genomes using STAR version 2.7.11b [41] to recover strain-specific transcripts and improve mapping accuracy through the detection of spliced reads and exon–exon junctions. For MetaT we used the STAR functions: —sjdbGTFfile target.gtf to include genome annotations; —outSAMtype BAM SortedByCoordinate to generate sorted bam outputs; —alignIntronMax 2000 to restrict maximum intron length, optimized for small genomes. Following alignment, both MetaG and MetaT datasets were subjected to quality filtering. Only those reads exhibiting 95% identity or higher identity and at least 80% aligned length were retained. In addition, sequences containing <30% high-complexity bases, or >75% low-complexity bases were discarded using bamFilters (https://github.com/institut-de-genomique/bamFilters). These filtering parameters, established in a previous study on *O. tauri* populations [42], were applied to minimize non specific alignments.

In total, we analyzed 295 samples (two different size-filters 0.8–5 μm and 0.8–2000 μm, and from two water column depths –surface and deep chlorophyll maximum—DCM) collected from 133 TO/TOPC stations. For downstream analyses, only samples with a genome mean depth coverage of ≥4× were retained.

Horizontal and vertical genome coverage were estimated using the samtools depth function. Per-base read depth across the genome was extracted with the -a option, and mean coverage depth was calculated as the sum of per-base depths divided by the total number of genomic positions. Breadth of coverage (≥4×) was defined as the proportion of genomic positions supported by at least four reads.

Gene-level coverage was assessed using the samtools coverage function, applying the -r option to restrict analysis to circadian clock and photoreceptor gene loci. This yielded mean depth and coverage breadth for each target gene. To account for differences in sequencing depth and gene length, read counts were normalized using the reads per kilobase per million mapped reads (RPKM) metric.

All statistical analyses and visualizations were performed in R (version 4.3.1; R Core Team, 2023). Data manipulation and wrangling were conducted using the tidyverse collection of packages [43], including dplyr, readxl, and writexl for reading and exporting Excel files. Correlation matrices were calculated using the base cor() function, and visualized using corrplot [44] and ComplexHeatmap [45]. Venn diagram was obtained with ggVennDiagram [46]. Enhanced figure annotations and formatting were carried out using ggplot2 [47].

The code used to perform all analyses are available at GitHub: https://github.com/jrigbr/ClimaClock.

### *Ostreococcus* sp*.* RCC809 TOC1 sequencing

To confirm the truncation of the CCT domain in *Ostreococcus* sp. RCC809 TOC1 sequence, we performed PCR amplification of the surrounding genomic region. Reaction was performed in 50 μL final volume containing 50 ng of *Ostreococcus* sp. RCC 809 genomic DNA, 5 μL of 5x SuperFi buffer, 5 μL of SuperFi GC Enhancer, 2.5 μL of each primer (10 μM), 1 μL of dNTP (10 mM) (Promega Corporation, USA), 0.5 μL of Platinum SuperFi DNA polymerase (Invitrogen, Thermo Fisher Scientific, USA), and water. The primer set used in this study was custom-synthesized by Eurofins (France) and listed in Table S4. The 778 bp amplified fragment was purified on agarose gel using NucleoSpin Gel and PCR Clean-up kit (Macherey-Nagel GmbH & Co. KG, Germany) and sequenced by Sanger technology (GENEWIZ GmbH, Germany) using the same primers set. Amplification began with a manual hot start step and an initial denaturation step of 95°C for 2 min followed by 35 cycles of 95°C for 5 s, annealing at 72°C for 1 min, extension at 68°C 1 min/kb and a final extension step for 10 min.

### Luciferase reporter lines construction and rhythmicity analysis

To explore whether circadian rhythmicity is maintained in the *Ostreococcus* sp. RCC809, luciferase reporter lines were established and their rhythmic behavior was examined. Experiments were carried out on *Ostreococcus* sp. strain RCC809 and *O. tauri* strain RCC745, both obtained from the Roscoff Culture Collection (https://roscoff-culture-collection.org/). Long mosaic constructs containing the full *CCA1* or *TOC1* clock genes, including promoter and coding sequence fused in frame with the CDS of firefly luciferase and the KanMX selection gene under the control of the Histone H4 promoter*,* were fused using a standard protocol of fusion PCR [48]. CCA1-luc and TOC1-luc initial fragments were amplified by PCR from the pOtLuc vector [14]. *Ostreococcus* sp. RCC809 CCA1 and TOC1 sequences were amplified directly from genomic DNA. The DNA fragment containing the firefly luciferase coding sequence and the KanMX CDS under the control of the histone H4 promoter was amplified from the cloning vector plasmid pOtLuc (Genbank accession number FN554877). Two chimeric long primers containing at the 5′ end, 60 nt-long complementary sequences were used to fuse the two fragments in the second PCR amplification (Table S4). PCR amplification reactions of initial fragments were performed in 50 μL final volume containing 1 μL of genomic DNA or plasmid (30 ng/μL), 5 μL of 10x buffer III, 10 μL of betain 5 M (Sigma-Aldrich, Germany), 1 μL of each primer 10 μM, 2.5 μL of dNTP 10 mM (Promega, USA), 0.5 μL of Platinum Pfx DNA polymerase (Invitrogen, USA) and water. Amplification began with a manual hot start step and an initial denaturation step of 95°C for 2 min followed by 35 cycles (95°C for 5 s, annealing at 72°C for 1 min, extension at 68°C 1 min /kb) and a final extension step for 10 min. The amplified products were purified from agarose gel using NucleoSpin Gel and PCR Clean-up kit (Macherey Nagel, Germany). For the fusion of the 2 initial fragments, long fusion PCR constructs were amplified using nested primers and the Expand Long Template PCR System (Roche, Switzerland). PCR amplifications were performed as described above in 50 μL final volume containing equimolar of each fragment previously purified (50 ng/kb) and 2 flanking nested primers (Table S4). Amplified DNA was purified as described above, precipitated with 2 volumes of ethanol and resuspended in water at a 1 μg/μL final concentration. The genetic transformation of *O. tauri* and *Ostreococcus* sp. RCC809 strains were performed by electroporation, using 5 μg of PCR product at 1 mg/ml concentration and transformants were selected in low-melting agarose plates containing G418 antibiotic (Sigma-Aldrich 1 mg/ml) as previously described [14]. Clones resistant to G418 were refreshed in 96 well microplates on screened on the basis of luminescence levels. For luminescence recordings, cells grown in culture medium with luciferin (20 μM) at a final density of 10 × 10^6^ cell/ml, were entrained under 16:8 h or 8:16 h light:dark (L:D) cycles (20 μmol·quanta·cm^−2^·s^−1^) for 4 days before being released into constant light (L:L) at 10 μmol·quanta cm^−2^·s^−1^ for 4 days. Luminescence was automatically recorded each hour using a Berthold Centro Luminometer [21]. Luminescence rhythms of luciferase reporter lines were analysed using Biodare2 [49]. Circadian rhythmicity was first assessed under free conditions of constant light using both the empirical JTK-CYCLE (eJTK) and Fast Transform Non-Linear Least Square Algorithm (FFT-NLLS) methods [50, 51]. Lines were considered as rhythmic for *P* < .0001 in eJTK analysis and relative amplitude errors (RAEs) <0.3 in FFTNLS analysis as previously described [14]. FFTNLS was used to determine Phases corresponding to acrophases (related to the Dark/Light transition) and Amplitude in LD conditions, and periods under constant light corresponding to free running conditions.

## Results and discussion

### In silico identification of additional putative circadian clock, light-sensing, and photoperiodism genes in Ostreococcus tauri

Through a comprehensive genomic screening of the seven Mamielalles genomes available in public databases, we aimed to identify putative master clock components, photoreceptors, and output pathways involved in the regulation of photoperiodism in these marine pico-green algae (Fig. 1, Tables S1 and S2).

Previous studies on *O. tauri* detected key core oscillator components, such as TOC1 and CCA1, alongside blue and green light-responsive photoreceptors like LOV-histidine kinases and Rhodopsin histidine kinases, the sole histidine kinases detected in *O. tauri* genome (Fig. 1A). Unlike plants’ PRRs, TOC1 receiver domain contains conserved asparte phosphorylation sites, suggesting that *O. tauri* TOC1 is a functional response regulator [14]. Functional evidences and mathematical modelling suggest that the two histidine kinases mediate green and blue light input to TOC1, a key response regulator in the two-component signaling (TCS) pathway [14, 19–21]. A histidine phosphorelay (HPT), typical of Histidine-Aspartate TCS may act as an intermediate in this input pathway.

In addition to TOC1, we identified in this study four other putative response regulators with receiver domains linked to various effector domains in *O. tauri* genome: a MYB GARP transcription factor (REC-GARP), WD40 (REC-WD40), Calmodulin kinase (REC-CAMK), and DNA-binding with one finger (REC-DOF) (Fig. 1A and B). GARP domains, alone or associated to REC domains MYB, are transcription factors found in master clock components (Lux) and regulators of photoperiodism (Ehd1) in plants [52, 53]. However, to our knowledge, WD40 and Calmodulin kinase effector domains have not been reported as plant response regulators. DOF domain proteins, known to regulate CONSTANS in plants and green algae [54, 55], suggest a potential link to photoperiodic control in algae. Additionally, we identified three putative CONSTANS-like proteins with CCT domains, including a newly annotated COL3 (ostta07g00980) (Fig. 1B, Tables S2 and S3). The CCT domain is a highly conserved 43-amino-acid motif involved in light signal transduction and nuclear localization [25, 56].

### Conserved and strain-specific genes across Mamiellales

Expanding our search to other Mamiellales species sampled in different latitudes (Fig. 1B, Fig. S1), including *Bathycoccus prasinos*, *Ostreococcus* spp., and two *Micromonas* spp., we identified 13 genes present across all genomes (Fig. 1B— All Strains box). These included five photoreceptors (CRY1, CRY DASH, Phototropin, LOV-HK, Rhod-HK), a histidine phosphorelay (HPT), the CCA1 master clock gene, three putative response regulators (REC-GARP, REC-WD40, REC-CAMK), and three COL/CMF. In addition to these shared genes, our analysis revealed lineage-specific gene losses and gene family expansions (Fig. 1B—Strains Specific box), often associated with innovations such as protein domain deletion or truncation. For example, *M. pusilla* uniquely encodes a phytochrome (PHY), a photoreceptor involved in red and far-red light perception in plants [57, 58]. Phylogenetic analyses conducted elsewhere indicate that the phytochrome detected in *M. pusilla* derives from an ancestral Archaeplastida lineage, predating the split between prasinophytes and streptophytes, rather than resulting from horizontal gene transfer [57].

**Figure 2:**
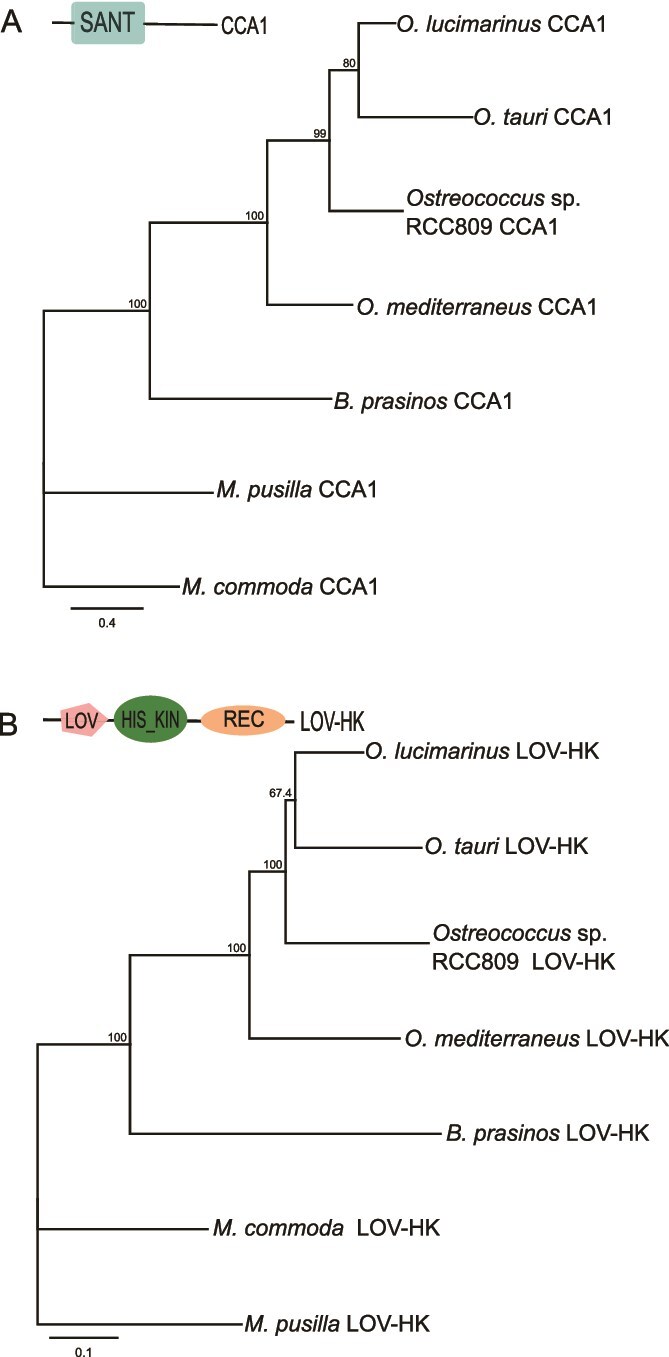
Phylogeny of core circadian clock related genes in Mamiellophyceae. (A) Circadian clock core gene *CCA1*. (B) LOV histidine kinase photoreceptor mediating blue light input to the clock. Trees were constructed based on the complete coding sequences available in Table S3, using maximum likelihood criterion performed with RAxML method on an amino acid alignment of proteins, and GAMMA DAYHOFF protein model. Bootstrap of 500 analysis higher than 70% are shown for each node. Protein conserved domains are showed: MYB SANT (SANT), receiver (REC), histidine kinase (HK), and light-oxygen-voltage (LOV). The scale bar represents amino acid substitutions per site.

CCA1 and LOV-HK phylogenies exhibited a topology consistent with that derived from ribosomal RNA gene phylogeny [59], indicating the monophyly of the Mamiellales clade, with *Micromonas* as the sister genus to the Bathycoccaceae, which includes *Bathycoccus* and *Ostreococcus* strains (Fig. 2A and B, Fig. S2). Similar topologies were observed for REC-CAMK, REC-GARP, and Phototropin (Fig S3A–C). This lends support to the hypothesis that the majority of the clock and light/photoperiod sensing genes originated early in the history of this order (Archaeplastida origin) and underwent vertical transferring during the process of speciation.

### C‌CT domain diversification in Mamiellales

**Figure 3:**
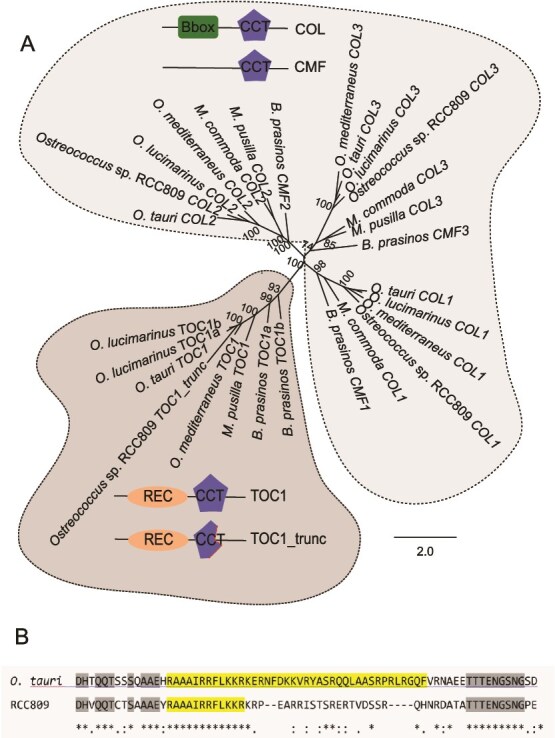
Evolutionary analysis of CCT domain family proteins: (A) Phylogenetic tree inferred using maximum likelihood criterion performed with RAxML method for the CCT domain family genes *TOC1* and *COL/CMF*, and GAMMA DAYHOFF protein model, represented as midpoint rooted tree. Bootstrap of 500 analysis higher than 70% are showed for each node. (B) CCT domain alignment showing truncation observed in *Ostreococcus* sp. RCC809 compared to *O. tauri.* Conserved CCT domain and conserved flanking regions are highlighted in yellow and grey respectively, ^*^indicate conserved amino acids. Conserved domains: Receiver (REC), CONSTANS, CONSTANS-like, TOC1 (CCT), zinc-finger B-box (B-box). The scale bar represents amino acid substitutions per site. Color circles highlights the TOC1 and CCT families’ clades.

The phylogeny of the CCT domain superfamily delineated two primary clades: the TOC1 and COL/CMF (Fig. 3A). TOC1 sequence exhibiting the highest divergence originates from *Ostreococcus* sp. RCC809, as evidenced by its placement on a long branch within the phylogenetic tree (Fig. 3A). The CCT domain in this sequence is truncated, and only its first 12 amino acids are present, while the remainder of the domain is missing. To avert misinterpretation due to genome assembly bias, we confirmed this truncation through Sanger sequencing in this study (Fig. 3B). The two *O. lucimarinus* TOC1 CDS share 100% nucleotide identity, indicative of a recent gene duplication event postdating *O. lucimarinus* speciation. In contrast, the two *B. prasinos* TOC1 CDS sequences share 35.6% nucleotide identity and 37.5% amino acid similarity (Blosum90 – Table S5), suggesting a more ancient duplication event and placement at the base of the TOC1 clade.

Divergence in COL/CMF proteins, which regulate circadian outputs, was genus-specific. *Ostreococcus* ssp. and *M. commoda* harbors three COL copies, whereas *M. pusilla* has two copies and *B. prasinos*, instead possess three CMF-like proteins (Figs 1 and 3A, Tables S2 and S3) [27, 60]. The central distinction between these two classes of CCT-containing proteins lies in the presence of a zinc-finger B-box domain in COL, conspicuously absent in CMF. This differentiation, well-documented in plants, particularly cereals [61, 62], is intriguingly observed. *Ostreococcus* and *Micromonas*-COL and *B. prasinos*-CMF clustered closely together, forming three sister clades despite the divergence in the zinc-finger B-box domain. This observation implies the orthology of COL and CMF in Mamiellales. These gene duplications, possibly arising from transpositional or whole-genome duplication events, may have contributed to the evolution of photoperiodic regulation in these species, similar to the role of CONSTANS in plant flowering [63]. The loss of the zinc-finger B-box domain in *B. prasinos* likely result independently across the three copies given the dissimilarity observed specially in the amino end region. The CONSTANS family multicopies are also common in plants, *Arabidopsis thaliana* containing 17 COL-like sequences showing one or two zinc-finger B-box domains. Analogous to plants, where CONSTANS, among other genes, orchestrates flowering control in response to the annual long-day photoperiodic cue, this observation prompts us to speculate that the COL/CMF genes in Mamiellalles could similarly play a role in modulating seasonal events such as algal blooms. This may be triggered by an optimal interplay of environmental conditions intricately linked to photoperiodic and temperature cues as evidenced in highly reoccurring winter blooms of *Bathycoccus* and *Micromonas* in long term time series [4].

### Diversity of photoreceptors duplications in Mamiellales

**Figure 4:**
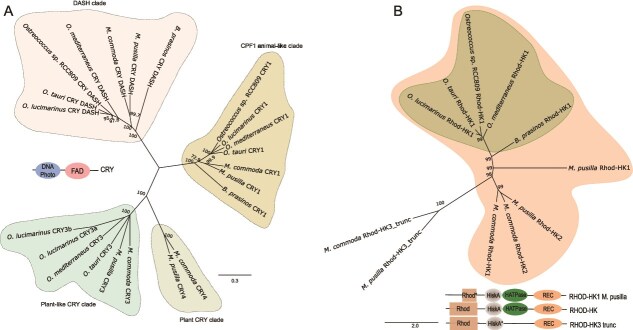
Diversity of Cryptochrome and Rhodopsin photoreceptors in Mamiellophyceae phylogeny of (A) Cryptochromes (CRY) and (B) Rhodopsin histidine kinases (Rhod-HK) families using maximum likelihood criterion performed with RAxML method on an amino acid alignment of proteins, and GAMMA DAYHOFF protein model, represented as midpoint rooted tree. Bootstrap of 500 analysis higher than 70% are showed for each node. Conserved domains: DNA photolyase (DNA photo); FAD binding domain (FAD); Rhodopsin (Rhod); histidine kinase A (HiskA); ATPase domain (HATPase); receiver (REC). The scale bar represents amino acid substitutions per site. Different colors in Cryptochrome tree highlight clades of CRY-family and in Rhod-HK highlight the Bathycoccaceae family phylogeny in green and the Mamielalles clade in orange.

Cryptochromes are ubiquist blue light photoreceptors but are also involved in the core circadian clock machinery of mammals. In *O. tauri*, three types of Cryptochromes have been identified (Figs 1 and 4A), including CPF1 (CRY1), phylogenetically related to animal like 6–4 photolyase, CRY DASH a family of Cryptochromes found in both eukaryotes and prokaryotes, and CRY3 a plant like Cryptochrome (CRYP) to that of the diatom *P. tricornutum* which retained a photolyase activity [18, 64]. Whereas CRY DASH and CRY1 were found in all Mamiellales, CRY3 was detected in *O. tauri, O. mediterraneus*, two copies in *O. lucimarinus* (CRY3a, CRY3b) and *Micromonas* spp. with an additional plant Cryptochrome (CRY4) exclusive to *Micromonas* spp. (Figs 1, 4A).

Gene duplication events, which can lead to neofunctionalization or specialization by sub-functionalization [65, 66] were evident in the phylogeny of the green light photoreceptor Rhod-HK. The Rhod-HK tree comprises two clades mirroring the Mamiellales phylogeny, one clade corroborates with the Bathycoccaceae family phylogeny, and another comprising *Micromonas* duplications located outside this clade (Fig. 4B). We posit that Rhod-HK duplications arose post *Micromonas* divergence. *M. pusilla* possesses a truncated Rhod-HK1 with a shorter Rhodopsin domain, though positioned in a long branch in the phylogenetic tree. Furthermore, both *M. commoda* and *M. pusilla* possess an extra Rhod-HK (Rhod-HK3) which harbors a domain with limited homology to HisK signal transduction histidine kinase sequence (dimerization/phosphoacceptor domain) lacking the Histidine kinase ATPase (HAPTase) domain. As the HisK domain is known to undergo autophosphorylation, followed by phosphotransfer to a partner response regulator (RR), in response to external stimulation of the effector domain (i.e Rhodopsin by green light), the functionality and role of this Rhod-HK variant observed here remains to be addressed.

### Geographical distribution and regulatory divergence of circadian clock-related genes in Mamiellales

To investigate the geographical distribution, expression regulation, gene duplication, and protein sequence variations in Mamiellales’ circadian clock-related genes, we explored metagenomic and metatranscriptomics data obtained from the TOs and the TOPC projects. This involved aligning the recovered reads to the seven reference genomes of Mamiellales. Previous studies have documented the widespread abundance of *B. prasinos*, *Ostreococcus* sp. RCC809, and *M. commoda* in oceans [67]. However, the oceanic distribution of *M. pusilla*, *O. tauri, O. mediterraneus,* and *O. lucimarinus* remain less known due to the lower abundance and restriction of these species to a few locations [67]. Building upon this information, we explored variations of candidate genes in the most represented species, *B. prasinos*, *M. commoda*, and *Ostreococcus* sp. RCC809.

**Figure 5:**
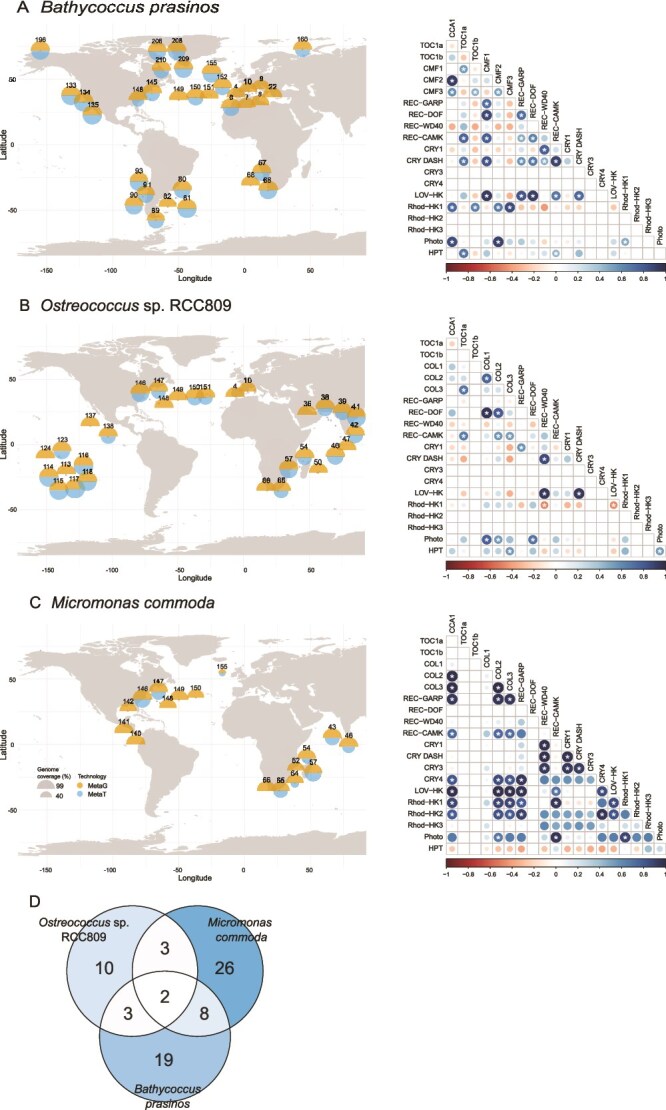
Oceanic distribution and co-expression patterns of circadian clock and light sensing genes in Mamiellophyceae: (A) *B. prasinos*, (B) *Ostreococcus* sp. RCC809 and (C) *M. commoda*. Maps illustrates the oceanic occurrence of strains represented in circles by genome coverage percentage calculated by metagenomics (MetaG-orange circles) and metatranscriptomics (MetaT-blue circles) reads recruited from Tara oceans and Tara polar circle expeditions. Numbers on top of the circles are Tara station IDs. Correlation plots were calculated by Pearson coefficient of circadian gene expression RPKM patterns in ocean obtained by MetaT analysis. Positive correlations are displayed in blue and negative correlations in red color, white-stars represent correlations with *P* < .05. Color intensity and the size of the circle are proportional to the correlation coefficients. (D) Venn diagram showing pairs of genes with significant correlations shared between strains.

The samples were selected after filtering for genomes with a mean depth greater than fourfold coverage, with overall mean coverage breadth ranging from 80% to 99% (Fig 5A–C). In total, we analyzed samples from 33 stations for *B. prasinos*, 30 stations for *Ostreococcus* sp. RCC809, and 17 stations for *M. commoda.* (Fig 5A–C). These samples spanned Polar Regions (Arctic), temperate/subtropical (northern and southern) latitudes, and one station in the Southern Ocean, along with warmer, low-latitude waters (Fig 5A–C). While *B. prasinos* was detected in polar, temperate and southern latitudes; *M. commoda*, and *Ostreococcus* sp. RCC809 were restricted to low latitudes regions, consistent with previous findings [67].

The presence of the target genes was confirmed in all the metagenomic samples that passed the fourfold genome depth filter, regardless of species, thus dismissing the hypothesis of gene deletion in population variations linked to geographical location (Figs S4–S6). Gene copy variants with high similarity that could bias the mapping were found to be negligible in the evaluated strains, with the exception of TOC1 in *O. lucimarinus*. Therefore, the observed variation in RPKM values among genes at different sampling stations reflects environmental differences in species abundance rather than gene copy number variation.

Given the critical influence of seasons and day time on light sensing and circadian clock gene expression [68], the sampling protocol established in the TOs project prevented us from making direct comparisons of gene expression across stations. In several cases, MetaT mapping did not pass the minimum cut-off threshold, or transcripts were absent for genes detected in the metagenomic data (Figs S4–S6), suggesting that these genes were not expressed at the time of sampling (e.g. in O. tauri, TOC1 transcripts peak at night but are minimal at dawn).

Despite these constraints, examining the co-expression patterns of candidate genes within individual species provides valuable insights into their potential functional divergence, even in the absence of strong cross-species conservation. Duplicated genes such as *B. prasinos TOC1a*/*TOC1b* and the three *Rhod-HK* genes of *M. commoda* exhibit weak and statistically nonsignificant co-expression (Fig 5A–C), reinforcing the interpretation that these paralogs lack tightly coordinated expression profiles. Such a lack of correlation supports the hypothesis that they may have undergone sub-functionalization or neofunctionalization, resulting in distinct regulatory roles or environmental responses within each species.

Comparisons between species further revealed differences in co-expression correlations. *B. prasinos* and *M. commoda* shared 10 pairs of co-expressed clock-related genes, whereas *B. prasinos* shared only 5 pairs with *Ostreococcus* sp. RCC809 (Fig. 5D), despite *Bathycoccus* and *Ostreococcus* being more closely related phylogenetically (Fig. S2). For instance, we found positive correlations in the expression of *CMF2/COL2* and *CMF3/COL3*, as well as *CCA1* and *CMF2/CMF3/-COL2/COL3*, common in both *B. prasinos* and *M. commoda* (Fig 5A and C). Similarly, co-expression between *CCA1* and *Rhod-HK1* was observed in *B. prasinos* and *M. commoda*, while these associations were not conserved in *Ostreococcus* sp. RCC809 (Figs. 5A–C). The expression of *REC-DOF*, a putative regulator of CONSTANS, also differed, it correlated positively with *CMF1* in *B. prasinos*, with *COL1* and *COL2* in *Ostreococcus* RCC809, while *REC-DOF* was absent from *M. commoda*. Across all three genera, only two genes co-expressed pairs were consistently observed (REC-WD40 and CRY DASH; COL2/CMF2 and Phototropin) pointing to a small but potentially essential set of conserved components (Fig. 5D). Their dual roles as photoreceptors and transcriptional regulators suggest these genes may form part of a core module in Mamiellales across diverse marine environments, highlighting new candidate genes supporting light/photoperiod response.

Technical limitations such as sequencing depth and sampling time remain significant constraints in projects using environmental metatranscriptomics. For instance, TOC1 and CCA1, known to be correlated under day/night cycles through a negative transcriptional feedback loop [14], were not correlated in our analyses. This likely reflects the fact that, in *O. tauri*, TOC1 transcript levels peak at night but remain minimal during the daytime, when TOs samples were collected. A more systematic identification of co-expressed gene clusters will require transcriptome analyses of Mamiellales species under varying day lengths, either *in situ* or under controlled laboratory conditions.

Overall, the distinct correlation patterns observed appear to be shaped by ecological and environmental factors beyond phylogenetic relatedness or geographic distribution. *B. prasinos* dominates in cold, high-latitude waters, whereas *Ostreococcus* sp. RCC809 and *M. commoda* are predominantly found in warmer, low-latitude environments. This raises the possibility that organisms inhabiting high latitudes, where seasonal and photoperiodic variations are more pronounced, may be more likely to retain or evolve robust circadian clock components compared to their low-latitude counterparts.

### Loss of the TOC1/CCA1 circadian oscillator in the low-latitude Mamiellales Ostreococcus sp. RCC809

An important consideration is the *TOC1* gene, a core component of the circadian clock in *O. tauri*, which shows remarkable variability among Mamiellales. It is the most divergent gene in our comparison, differing in presence/absence, copy number, and structural organization across species. *B. prasinos* possesses two potentially functional copies, TOC1a and TOC1b, with different expression patterns as inferred from the correlation results (Fig. 5A). TOC1 was absent from *M. commoda,* and truncated in the CCT domain of *Ostreococcus* sp. RCC809. We observed a stable and high coverage in the truncated portion of TOC1 in *Ostreococcus* sp. RCC809 in the TOs metagenomic dataset suggesting that this modification is present in oceanic populations of this strain (Fig. S7A and B). The truncation of more than half of the CCT domain in *Ostreococcus* sp. RCC809 suggests that the CCA1/TOC1 negative feedback loop at the heart of circadian clock in *O. tauri*, may be not functional in *Ostreococcus* sp. RCC809.

**Figure 6:**
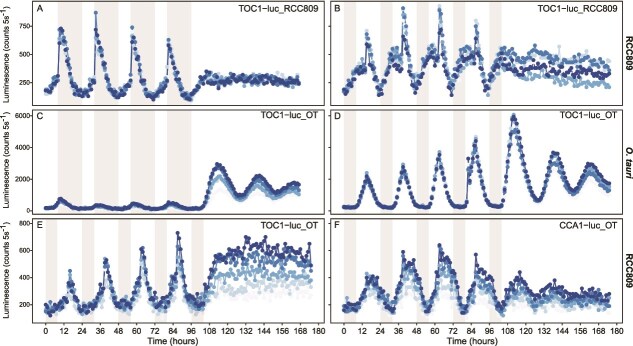
Functional analysis of *Ostreococcus* sp. RCC809 *TOC1* and *CCA1* circadian clock genes. *Ostreococcus* sp. RCC809 and *O. tauri* TOC1-luc/CCA1-luc were expressed in *Ostreococcus* sp. RCC809 and *O. tauri* under short-day (8:16 h L:D) and long-day (16:8 h L:D) for 96 h prior to be transferred to constant light condition. Expression of *Ostreococcus* sp. RCC089 TOC1-luc in RCC809 under constant light (L:L) after short-day entrainment (A) or long-day entrainment (B). Expression of *O. tauri* TOC1-luc_OT in *O. tauri* under constant light after short-day entrainment (C) and long-day entrainment (D). Heterologous expression of *O. tauri* TOC1-luc_OT in *Ostreococcus* sp. RCC809 in constant light after long day entrainment (E). Heterologous expression of *O. tauri* CCA1-luc_OT in RCC809 in constant light after long day entrainment (F). Grey bars indicate dark periods, and colored dotted-lines represent individual traces (*n =* 5). Rhythm’s analysis is detailed in Table S6.

To test whether the CCA1/TOC1 negative feedback loop is functional in *Ostreococcus* sp. RCC809, we generated luciferase translational reporters to monitor the expression of *CCA1* (CCA1-luc) and *TOC1* (TOC1-luc) genes. The complete genes, including promoter and coding sequences fused in frame to firefly luciferase were electroporated in *O. tauri* and *Ostreococcus* sp. RCC809 strains, following the methodology described in Corellou *et al.* [14]. We first introduced TOC1-luc sequences obtained from *Ostreococcus* sp. RCC809 (TOC1-luc_RCC809) into *Ostreococcus* sp. RCC809 cells. Under 8:16 h L:D (light:dark short-day) and 16:8 h L:D (light:dark long-day) cycles, we observed a peak in TOC1-luc expression at dusk followed by a sharp decrease (Fig. 6A and B, Table S6), suggesting a dark-dependent degradation of TOC1 in *Ostreococcus* sp. RCC809 as previously reported in *O. tauri* [19]. Circadian rhythms were analyzed under constant light (L:L) using both FFTNLLS and eJTK methods (See Methods section). Under constant light condition, the TOC1-luc_RCC809 line lost its rhythmic expression (Fig. 6A and B, Table S6). Additionally, the evening element (EE), a conserved 9-bp sequence present in the TOC1 promotor of all the others species, is absent from *Ostreococcus* sp. RCC809 (Table 1). This EE serves as a binding site for CCA1 protein and is critical in the TOC1 regulation in *O. tauri* [14]. These results indicate that while the diel control of TOC1 expression remains intact in *Ostreococcus* sp. RCC809, its circadian control has been lost, likely due to the nonfunctionality of TOC1/CCA1 feedback loop. We subsequently transformed *Ostreococcus* sp. RCC809 with the functional *O. tauri* TOC1-luc (TOC1-luc_OT) sequence, which lengthens the free running period of CCA1-luc in *O. tauri* [14, 21]. Similar to the previous result, the TOC1-luc_OT expression also displayed a peak at dusk under L:D 16:8 h with loss of rhythms under constant light (Fig. 6E, Table S6). Because TOC1-luc_OT promoter contains a functional EE, this leads to an alternative hypothesis: *Ostreococcus* sp. RCC809 CCA1 also have lost its ability to regulate the expression of TOC1 clock gene.

**Table 1:** Presence of evening element (EE) in the different strains evaluated in this study.

Strain	EE	Position
*B. prasinos* TOC1a	AAATATCT	−289(+)
*B. prasinos* TOC1b	Absent^*^	
*O. tauri*	AAAATATCT	−268(+)
*Ostreococcus* sp. RCC809	Absent^*^	
*O. lucimarinus*	AAAATATCT	−375(−)
*O. mediterraneus*	AAAATATCT	−784(+)
*M. pusilla*	AAAATATCT	−470(−)
*M. commoda*	§	

^*^Not observed within 2000 bp upstream of the start codon. (+) and (−) strands

§- *TOC1* gene is absent in *M. commoda.*

No luminescent lines were obtained when transforming *Ostreococcus* sp. RCC809 cells with *Ostreococcus* RCC809 CCA1-luc. However, we successfully transformed *O. tauri* CCA1-luc (CCA1-luc_OT) into *Ostreococcus* sp. RCC809 cells (Fig. 6F) and *Ostreococcus* sp. RCC809 CCA1-luc (CCA1-luc_RCC809) into *O. tauri* cells (Fig. S8A and B). When expressed in *Ostreococcus* sp. RCC809, CCA1-luc_OT showed no circadian rhythms in L:L after short day entrainment but a weak rhythmicity was detected in constant light condition after long day entrainment (Fig. 6F and Fig. S8, Table S6). However, when removing the first 24 h corresponding to the transition from L:D to L:L the eJTK method failed to detect rhythms in 4 out 5 lines (Table S6). In addition, CCA1-luc_OT peaked at dusk in 16:8 h L:D cycle (Fig. 6F, Table S6) i.e. 2 h earlier compared to wild type CCA1-luc_OT in *O. tauri* (Fig. S8C and D, Table S6) [14].

The lack or strong attenuation of circadian rhythms when expressing CCA1-luc_OT in *Ostreococcus* sp. RCC809 in constant light, is not surprising given that *Ostreococcus* sp. RCC809 TOC1 lacks a functional CCT domain. In contrast, CCA1-luc_RCC809 expression in *O*. *tauri* remains circadian under constant light suggesting that the promoter of *Ostreococcus* sp. RCC809 CCA1 contains regulatory sequences required in circadian regulation by *O. tauri* TOC1. However, CCA1-luc_RCC809 showed abnormal phases under conditions of long days and light–dark cycles with a peak during the day (i.e. 12 h before CCA1 in *O. tauri*), as well as a loss of circadian rhythms in constant light, suggesting that CCA1-luc_RCC809 is not fully functional with respect to the TOC1/CCA1 negative feedback loop (Fig. S8B, Table S6). Overall, our experimental data confirm that the CCA1/TOC1 feedback loop is not functional in *Ostreococcus* sp. RCC809.

The absence of TOC1 in *M. commoda* and the lack of a functional TOC1/CCA1 circadian oscillator in *Ostreococcus* sp. RCC809 lead us to speculate that Mamiellales inhabiting low latitudes, where day length and temperature remains relatively constant throughout the year, may not require a fully functional TOC1/CCA1 circadian oscillator to adjust their physiology to photoperiod changes. This inference is analogous to patterns described in the cyanobacterium *Prochlorococcus*, which lacks the central clock component KaiA yet retains timekeeping mechanisms in stable environments [69]. Conversely, organisms inhabiting temperate and polar regions such as *B. prasinos*, *O. tauri*, *O. lucimarinus*, *O. mediterraneus* or *M. pusilla* would rely on fully functional clocks to adjust their physiology to varying photoperiods along the year. Future studies should aim to directly test this hypothesis through controlled laboratory experiments, examining circadian rhythms and photoperiodic responses in tropical species such as *Ostreococcus* sp. RCC809 and *M. commoda*, as well as in higher-latitude Mamiellales strains, including *O. tauri* and polar strains of *B. prasinos*. Alternatively, targeted disruption of TOC1 in *O. tauri* via homologous recombination could provide precise insights into the specific role of TOC1, and more broadly the TOC1/CCA1 oscillator, in adaptation to changing photoperiods [70].

## Conclusion

Due to their wide geographical distribution, compact genomes, and shared photoreceptors and circadian clock components with Streptophyta and Chlorophycea, Mamiellales represent an intriguing group of phytoplankton for studying the diversity of photoperception and time-measurement mechanisms from both evolutionary and environmental perspectives. On the basis of the knowledge acquired in *O. tauri*, we propose that the circadian clock in Mamiellales is based on a two-component system that integrates the light signal into the TOC1/CCA1 central oscillator. Members of the CONSTANS/DOF-type photoperiodism genes, like in plants, may regulate the downstream response to photoperiod, which varies according to latitude and season. Here we show that there is an unexpected diversity of potential players involved in light/photoperiod sensing and circadian clock in these picoalgae. In the Mamiellaceae (genus *Micromonas*), certain photoreceptors, such as Rhodopsin histidine kinase, have undergone duplication events, potentially linked to neo- or sub-functionalisation. Within Mamiellales, the CCT domain proteins of the TOC1 and CONSTANS family are undoubtedly the most variable, with events such as TOC1 duplication or the loss of the B-box domain in *B. prasinos* CONSTANS. The absence of the central clock gene TOC1 in *M. commoda* or its truncation in *Ostreococcus* sp. RCC809, leading to a defect of the central TOC1/CCA1 oscillator, suggest that CCT domain proteins variation may be crucial to adaptation to latitudes, similar to adaptations observed in cultivated plants. We posit that low latitude phytoplankton species, which do not experience dramatic variations in photoperiod throughout the year, may not need a fully functional circadian clock or the CCA1/TOC1 oscillator may be specifically involved in photoperiod responses. Further physiological and genomic analysis of natural variants of the cosmopolitan species *Bathycoccus prasinos* may help to clarify these hypotheses in the future [71].

## Supplementary Material

Supp_Figure_1_wraf263

Figure_Supp2_wraf263

Figure_Supp3_wraf263

Figure_Supp4_wraf263

Figure_Supp5_wraf263

Figure_Supp6_wraf263

Figure_Supp7_wraf263

Figure_Supp8_wraf263

Supplementary_tables_Rigonato_et_al_wraf263

Supplementary_Material_legends_wraf263

## Data Availability

Reference genomes investigated here are available at Online Resource for Community Annotation of Eukaryotes database: https://bioinformatics.psb.ugent.be/orcae/ Tara Oceans and Tara Oceans Polar Circle raw metagenomic and metatranscriptomic data are available at European Nucleotide Archive (https://www.ebi.ac.uk/ena). All identifiers are in Supplementary Table S1_Data Availability.
