# Supplementary figures and images for: Latitudinal diversity in circadian and light-sensing genes in an ecologically vital group of marine picoeukaryote algae
Source: ISME J. 2025 Nov 28;19(1):wraf263. doi: 10.1093/ismejo/wraf263 (PMC12704426; doi:10.1093/ismejo/wraf263)

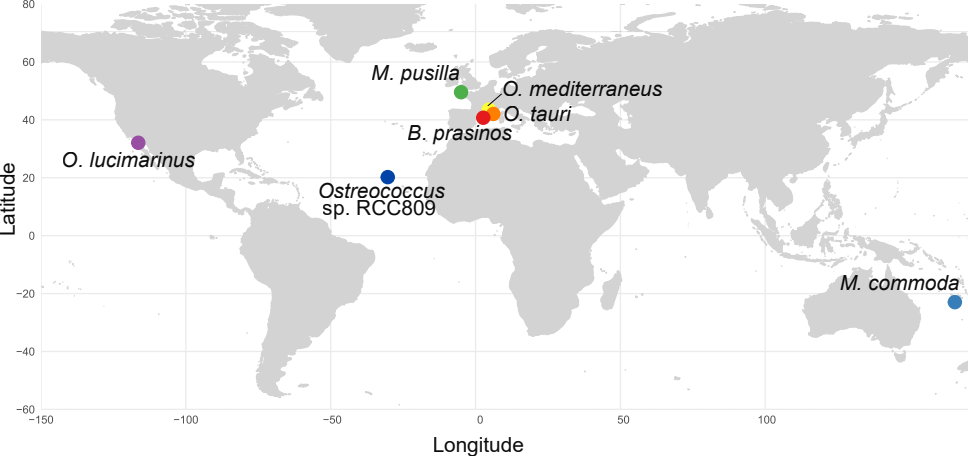

Supplement: Supp_Figure_1_wraf263 [file supp_figure_1_wraf263.pdf]

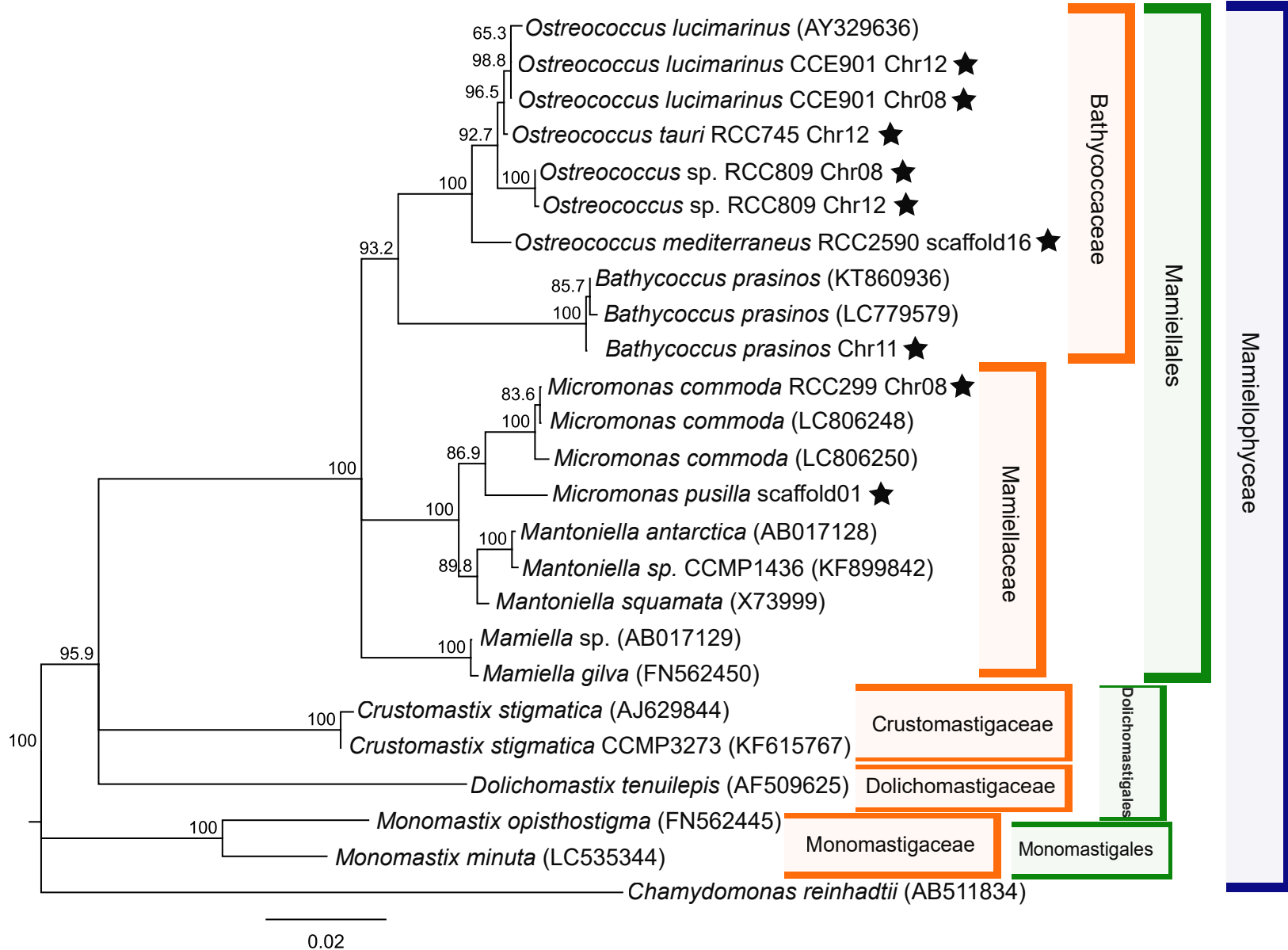

Supplement: Figure_Supp2_wraf263 [file figure_supp2_wraf263.pdf]

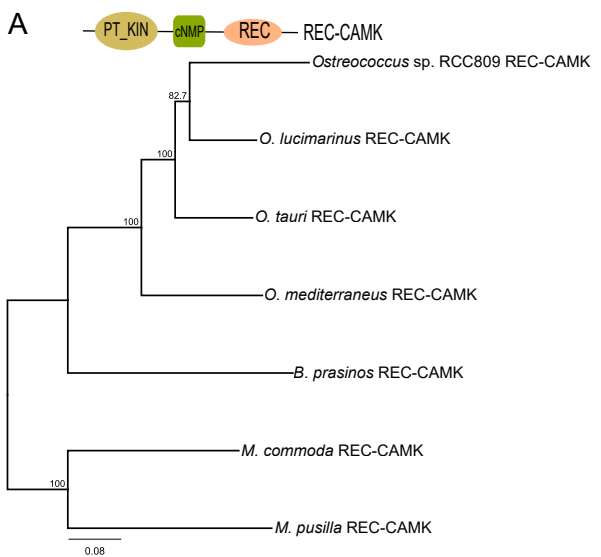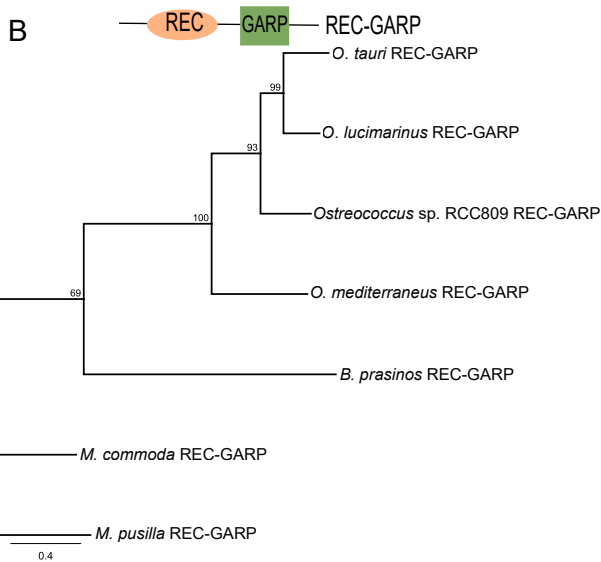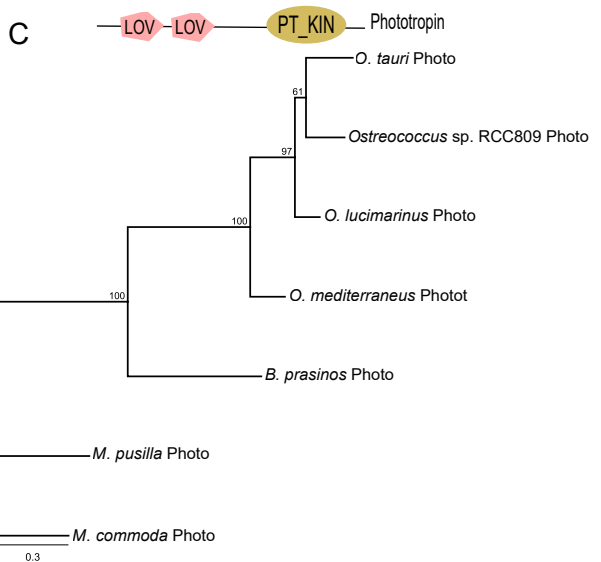

Supplement: Figure_Supp3_wraf263 [file figure_supp3_wraf263.pdf]

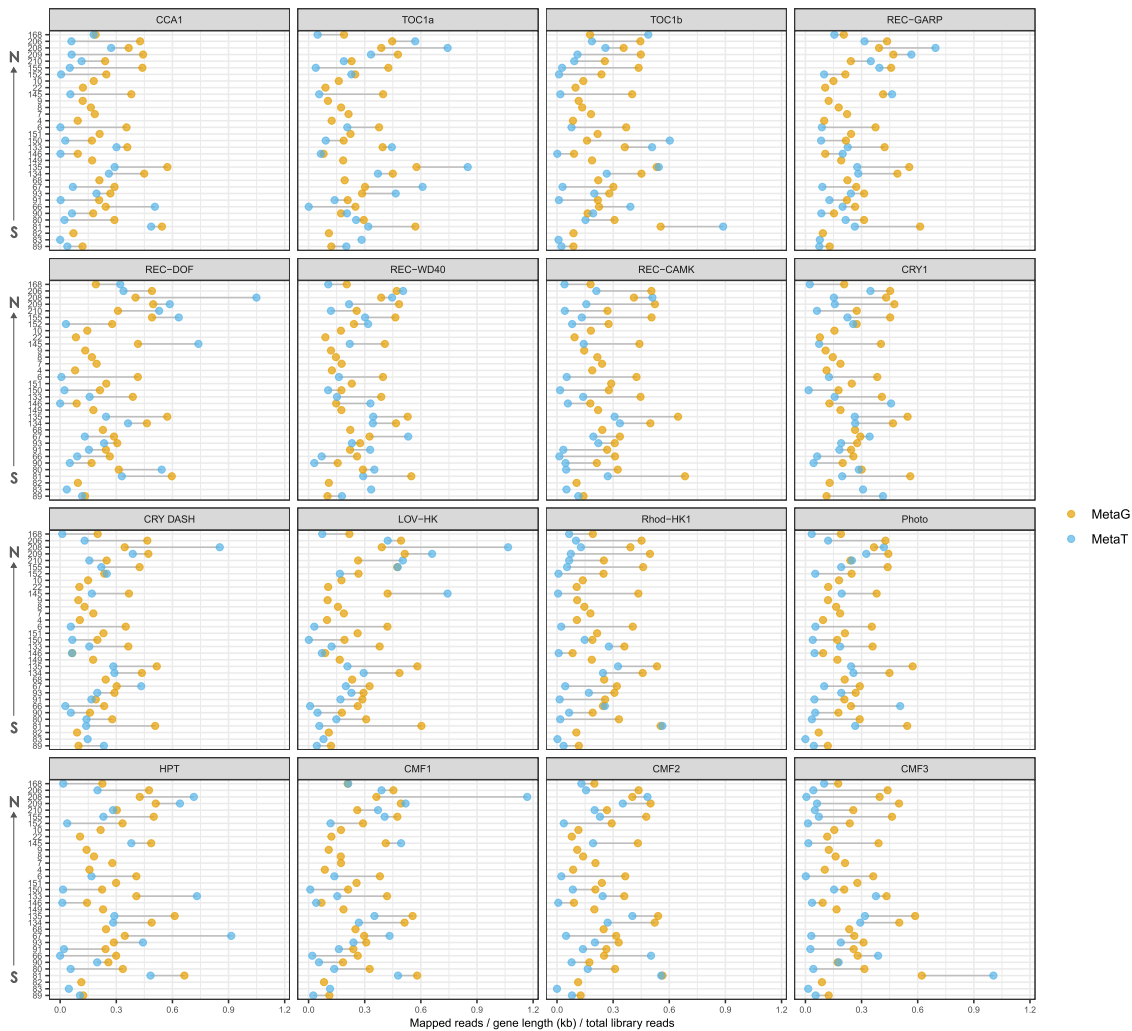

Supplement: Figure_Supp4_wraf263 [file figure_supp4_wraf263.pdf]

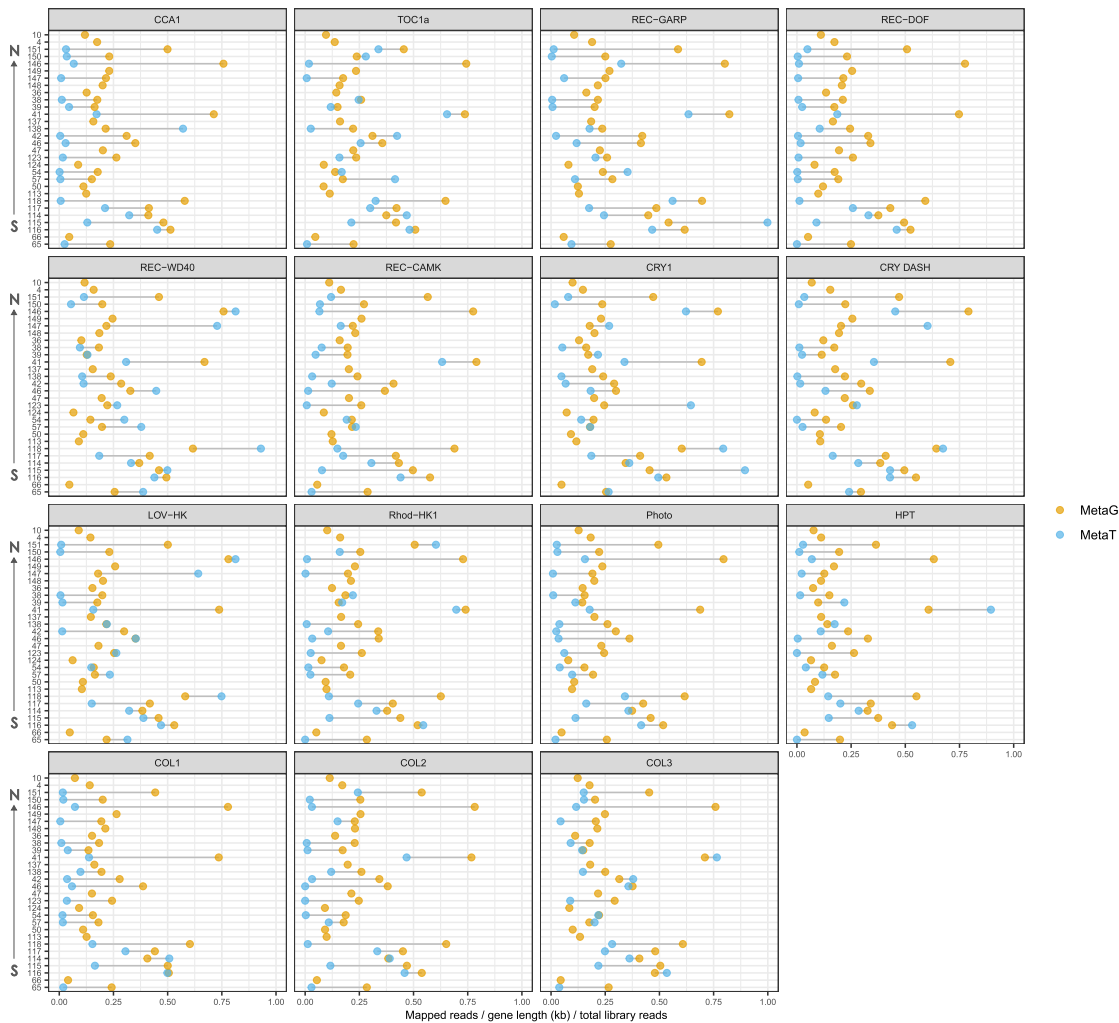

Supplement: Figure_Supp5_wraf263 [file figure_supp5_wraf263.pdf]

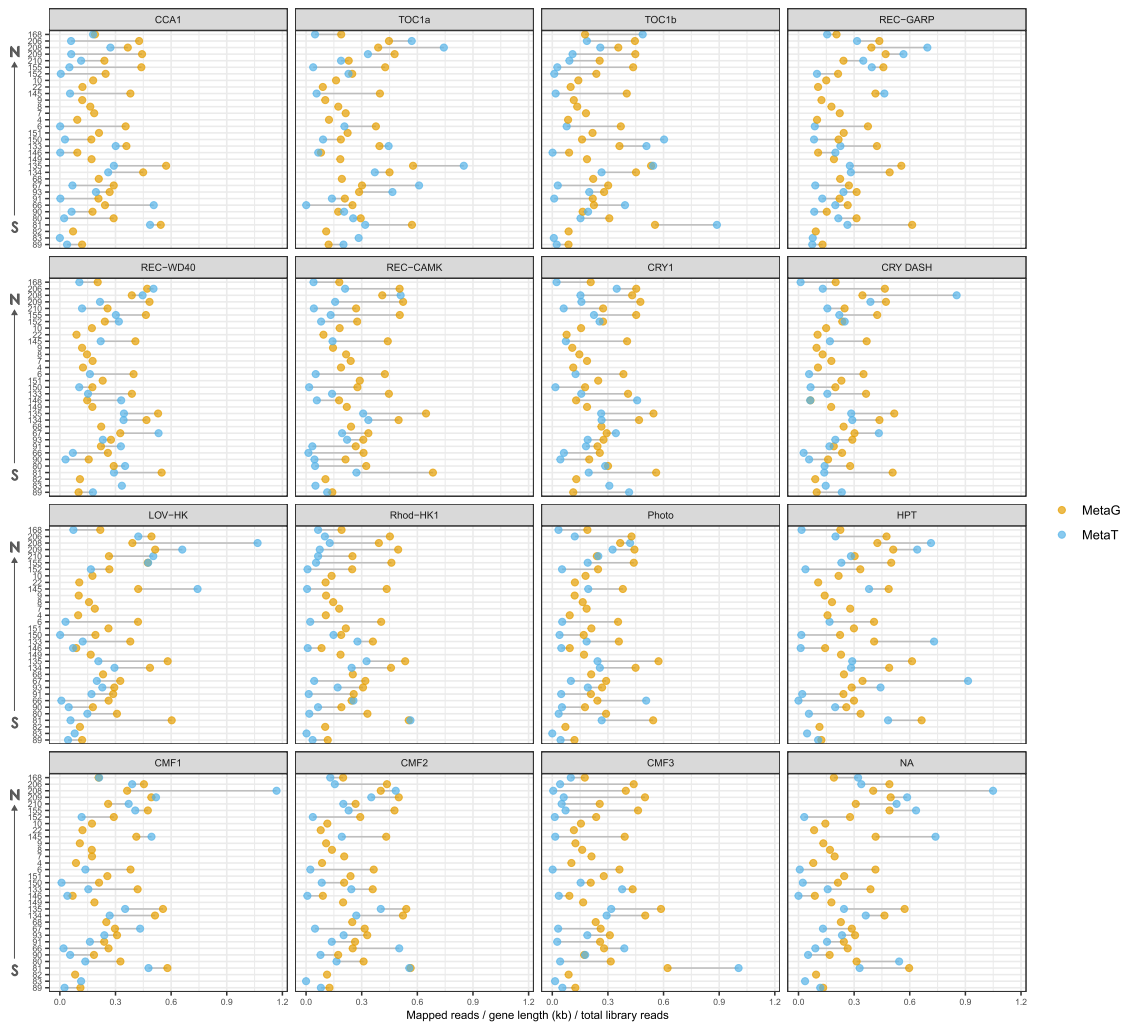

Supplement: Figure_Supp6_wraf263 [file figure_supp6_wraf263.pdf]

A

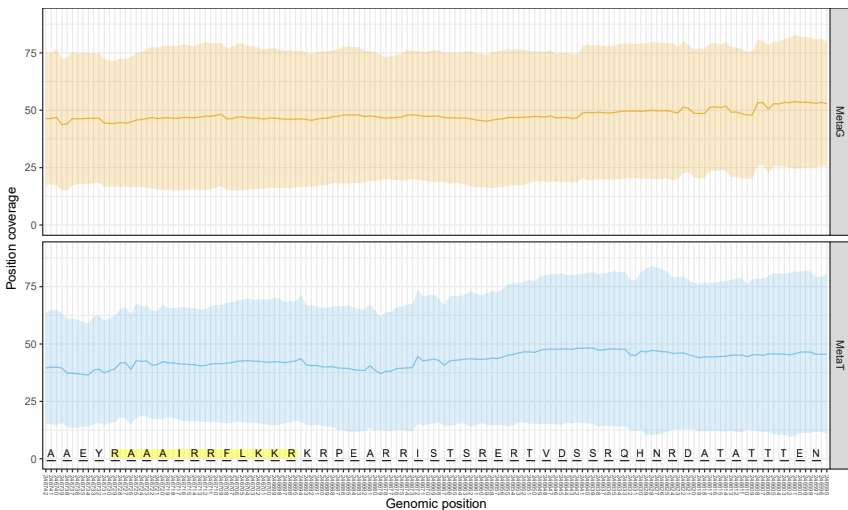

B

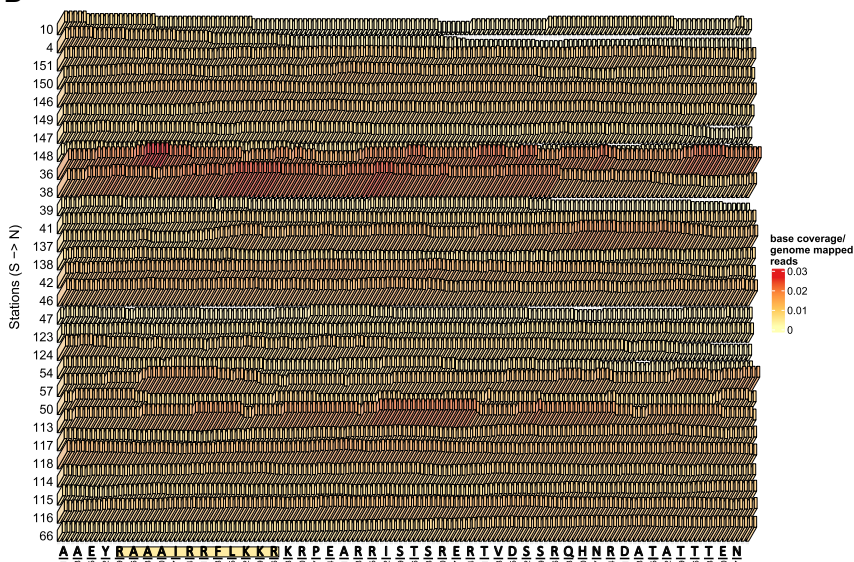

Supplement: Figure_Supp7_wraf263 [file figure_supp7_wraf263.pdf]

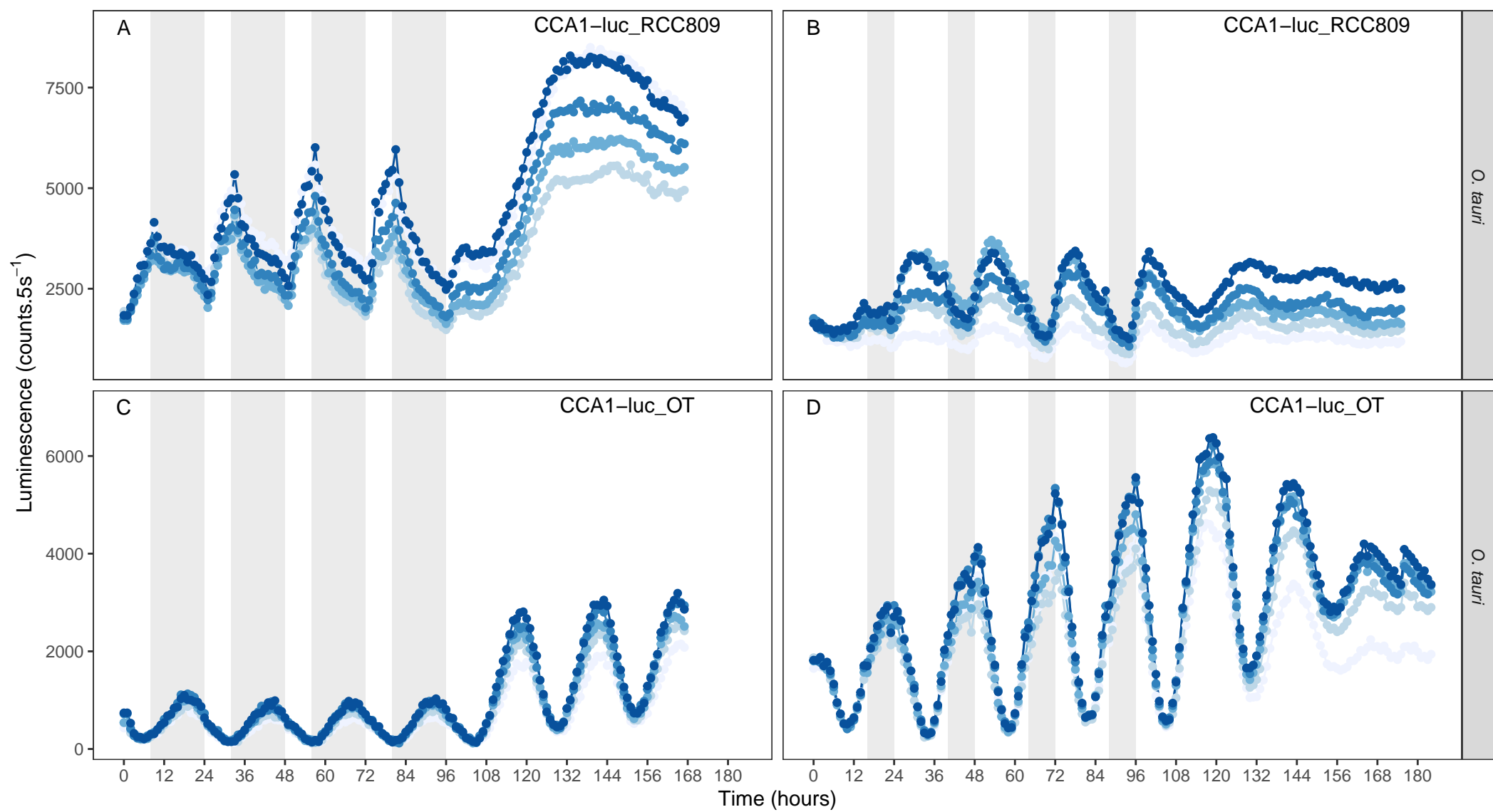

Supplement: Figure_Supp8_wraf263 [file figure_supp8_wraf263.pdf]
