# Supplementary material for: Latitudinal diversity in circadian and light-sensing genes in an ecologically vital group of marine picoeukaryote algae
Source: ISME J. 2025 Nov 28;19(1):wraf263. doi: 10.1093/ismejo/wraf263 (PMC12704426; doi:10.1093/ismejo/wraf263)
Supplement: Supplementary_Material_legends_wraf263 [file supplementary_material_legends_wraf263.docx]

**Supplementary Material:**

**Supplementary Table 1:** Data availability: URLs for Data Sets Utilized in This Study — Including Mamielalles Genomes, Tara Oceans Metagenomes and Metatranscriptomes

**Supplementary Table 2:** Gene list of the strains evaluated in this study: Gene name, Gene Domain and CDS

**Supplementary Table 3:** Gene Data Analyzed in This Study: Coding Sequences (CDS), Genomic Positions, Nucleotide Sequences, and Amino Acid Sequences

**Supplementary Table 4:** List of primers and DNA sources used for (1) construction of CCA1-luc and TOC-luc in *Ostreococcus tauri* and *Ostreococcus* spp RCC809, (2) amplification and sequencing of the CCT domain of TOC1-RCC809.

**Supplementary Table 5:** Pairwise BLOSUM similarity scores for CCT domain protein sequences

**Supplementary Table 6:** Rhythmicity analysis of TOC1 and CCA1 in *Ostreococcus sp.* RCC809 and *O. tauri* corresponding to luminescence traces shown in Figure 6 and Figure S8. Cells were entrained under long day or short day conditions for 96h before being released in constant light for 72 hours. Rhythms were analysed using FFTNLLS and EJTK methods using BIodare2 (<https://biodare2.ed.ac.uk/>). Phases (corresponding to acrophases) and Amplitude in L:D cyles were determined using FFTNLLS. Rhythmicity was assessed using both FFTNLLS and BD2EjTK in LD and LL conditions. Lines are considered as rhythmic (green color) for RAE <0.5 in FFTNLLS and pvalue < 0.0001 in eJTK analysis (true). Rhythmicity was also assessed using BD2ejTK after removing the first 24 hours in LL (116h to 167 h) corresponding to the transition from entraining to free running conditions.

**Supplementary Table 7:** Geographic coordinates (latitude and longitude) of Tara Oceans sampling stations**.**

**Supplementary Figure 1**. Geographic sampling locations of the strains analyzed in this study. The map indicates the latitude and longitude of the stations where these strains were collected.

**Supplementary Figure 2.** Neighbor-joining phylogenetic tree of Mamiellophyceae based on the 18S rRNA marker. The tree was generated in Geneious Prime using the Tamura–Nei nucleotide model, and the consensus tree was obtained after 500 bootstrap replicates. The class Mamiellophyceae comprises three orders: Mamiellales, Dolichomastigales, and Monomastigales. The strains evaluated in this study belong to two families within the order Mamiellales: Bathycoccaceae (*Bathycoccus prasinos* and *Ostreococcus* spp.) and Mamiellaceae (*Micromonas* spp.). Sequences obtained from genomes analyzed in this study are marked with stars, followed by the chromosome or scaffold identifier. Numbers in parentheses indicate the GenBank accession numbers of sequences retrieved from the database.

**Supplementary Figure 3:** Phylogeny of conserved light sensing and circadian clock related genes by maximum likelihood criterion performed with RAxML method on an amino acid alignment of proteins, and GAMMA DAYHOFF protein model constructed for A) REC-CAMK, B) REC-GARP C) Phototropin proteins. Bootstrap of 500 analysis higher than 60% are showed for each node. The scale bar represents amino acid substitutions per site. Domains: protein kinase (PT_KIN); cyclic Nucleotide Monophosphate binding domain (cNMP); GARP transcription factor (GARP); Light-Oxygen-Voltage (LOV).

**Supplementary Figure 4.** Occurrence and expression of light-sensing and circadian clock-related genes (boxes) from *Bathycoccus prasinos* across oceanic regions, based on recruitment of metagenomic (MetaG) and metatranscriptomic (MetaT) reads from Tara Oceans (TO) and Tara Polar Circle (TOPC) expeditions. The x-axis shows gene coverage calculated using the RPKM method (mapped reads / gene length [kb] / total library reads), representing gene abundance (MetaG) and expression level (MetaT). The y-axis indicates TO and TOPC station numbers, corresponding to those in the main Figure 5 maps and Supplementary Table S7, arranged by latitudinal gradient from south to north (S → N). Orange dots represent MetaG, blue dots represent MetaT, and grey bars indicate the difference in coverage between MetaG and MetaT.

**Supplementary Figure 5.** Occurrence and expression of light-sensing and circadian clock-related genes (boxes) from *Ostreococcus* sp. RCC809 across oceanic regions, based on recruitment of metagenomic (MetaG) and metatranscriptomic (MetaT) reads from Tara Oceans (TO) and Tara Polar Circle (TOPC) expeditions. The x-axis shows gene coverage calculated using the RPKM method (mapped reads / gene length [kb] / total library reads), representing gene abundance (MetaG) and expression level (MetaT). The y-axis indicates TO and TOPC station numbers, corresponding to those in the main Figure 5 maps and Supplementary Table S7, arranged by latitudinal gradient from south to north (S → N). Orange dots represent MetaG, blue dots represent MetaT, and grey bars indicate the difference in coverage between MetaG and MetaT.

**Supplementary Figure 6.** Occurrence and expression of light-sensing and circadian clock-related genes (boxes) from *Micromonas commoda* across oceanic regions, based on recruitment of metagenomic (MetaG) and metatranscriptomic (MetaT) reads from Tara Oceans (TO) and Tara Polar Circle (TOPC) expeditions. The x-axis shows gene coverage calculated using the RPKM method (mapped reads / gene length [kb] / total library reads), representing gene abundance (MetaG) and expression level (MetaT). The y-axis indicates TO and TOPC station numbers, corresponding to those in the main Figure 5 maps and Supplementary Table S7, arranged by latitudinal gradient from south to north (S → N). Orange dots represent MetaG, blue dots represent MetaT, and grey bars indicate the difference in coverage between MetaG and MetaT.

**Supplementary Figure 7:** Nucleotide coverage analysis of the TOC1 CCT domain truncation in *Ostreococcus* sp. RCC809. **A)** Average coverage curve (mean ± standard deviation) for nucleotide positions in Tara Oceans MetaG and MetaT datasets, spanning the genomic regions upstream and downstream of the CCT domain truncation. The x-axis represents the CCT amino acid sequence, with the conserved domain highlighted in yellow. **B)** Heatmap bar plot displaying nucleotide position coverage from the Tara Oceans metaG dataset, with individual rows corresponding to Tara Oceans sampling stations arranged by latitudinal geographic position. The x-axis represents the CCT amino acid sequence, with the conserved domain highlighted in yellow.

**Supplementary Figure 8:** Functional analysis of Ostreococcus sp. RCC089 CCA1 circadian clock gene. Ostreococcus sp. RCC809 and O. tauri CCA1-luc were expressed in Ostreococcus sp. RCC809 under short-day (8:16 h L:D), or long-day (16:8 h L:D) for 96 h prior to be transferred to constant light. Heterologous expression of Ostreococcus sp. RCC089 CCA1-luc in O. tauri under constant light after short-day entrainment (A) or long-day entrainment (B). Expression of O. tauri CCA1-luc in O. tauri under constant light after short-day entrainment (C) and long-day entrainment (D). Grey bars indicate dark periods, and colored dotted-lines represent individual traces (n=5). Rhythm’s analysis is detailed in Table S6.
